# Selected issues in sport-related concussion (SRC|mild traumatic brain injury) for the team physician: a consensus statement

**DOI:** 10.1136/bjsports-2021-104235

**Published:** 2021-06-16

**Authors:** Stanley Herring, W Ben Kibler, Margot Putukian, Gary S Solomon, Lori Boyajian-O'Neill, Katherine L Dec, R Robert Franks, Peter A Indelicato, Cynthia R LaBella, John J Leddy, Jason Matuszak, E Barry McDonough, Francis O'Connor, Karen Michelle Sutton

**Affiliations:** 1 Departments of Rehabilitation Medicine, Orthopaedics and Sports Medicine and Neurological Surgery, University of Washington, Seattle, Washington, USA; 2 Shoulder Center of KY, Lexington Clinic, Lexington, Kentucky, USA; 3 Princeton, Princeton, New Jersey, USA; 4 Colchester, Vermont, USA; 5 Overland Park Surgical Specialists & Sports Medicine, Overland Park, Kansas, USA; 6 Department of Physical Medicine and Rehabilitation, and Orthopaedic Surgery, Virginia Commonwealth University, Richmond, Virginia, USA; 7 Rothman Orthopaedic Institute, Philadelphia, Pennsylvania, USA; 8 University of Florida, Gainesville, Florida, USA; 9 Pediatrics, Northwestern University, Evanston, Illinois, USA; 10 Pediatric Orthopedics and Sports Medicine, Ann and Robert H Lurie Children's Hospital of Chicago, Chicago, Illinois, USA; 11 UBMD Orthopaedics and Sports Medicine, SUNY Buffalo, Buffalo, New York, USA; 12 Buffalo, New York, USA; 13 West VirginiaUniversity, Morgantown, West Virginia, USA; 14 Military and Emergency Medicine, Uniformed Services University of the Health Sciences, Bethesda, Maryland, USA; 15 Orthopaedic Surgery, HSS, New York, New York, USA

**Keywords:** concussion, brain, physician, sport, exercise

## Abstract

Selected Issues in Sport-Related Concussion (SRC|Mild Traumatic Brain Injury) for the Team Physician: A Consensus Statement is title 22 in a series of annual consensus documents written for the practicing team physician. This document provides an overview of selected medical issues important to team physicians who are responsible for athletes with sports-related concussion (SRC). This statement was developed by the Team Physician Consensus Conference (TPCC), an annual project-based alliance of six major professional associations. The goal of this TPCC statement is to assist the team physician in providing optimal medical care for the athlete with SRC.

## Introduction

Sport-related concussion (SRC) is a common injury managed by team physicians. This statement was developed by the Team Physician Consensus Conference (TPCC) to address selected issues on SRC relevant to the practicing team physician. Key points from this TPCC are noted below. This document is the latest revision to Team Physician consensus statements on SRC originally published in 2006^1^ and updated in 2011.^2^

**Key points**

- The diagnosis of sport-related concussion (SRC) remains a challenge due to non-specific symptoms and lack of objective biomarkers.
- SRC is a treatable condition.
- The number and severity of initial symptom burden is the best predictor for the duration of recovery.
- Current evidence suggests strict rest after SRC slows recovery and increases the probability of prolonged symptoms.
- The majority of athletes with SRC recover within a typical timeframe (2 weeks for adults and up to 4 weeks for children).
- Persisting Symptoms after SRC (PSaSRC) is defined as symptoms that last longer than the typical timeframe. The pathophysiology underlying PSaSRC is not entirely understood. It is thought PSaSRC is not caused by a single pathologic process, but rather an interaction of postinjury symptoms that are complicated by pre-existing, coexisting and/or resulting biopsychosocial factors.
- The management of disabling PSaSRC often requires a multidisciplinary approach.

## Methodology

The TPCC has been led by the American College of Sports Medicine (ACSM) Clinical Sports Medicine Leadership (CSML) committee for more than two decades. The TPCC was formed to create relevant, timely and condensed resources specifically for the team physician working with athletes at every level of competition.^3^ An executive committee of medical and orthopaedic team physicians from the CSML selects topics creates an outline based on their collective experience of the topic, then leads a delegation composed of two representatives from each of six major professional medical organisations including the: American Academy of Family Physicians, American Academy of Orthopaedic Surgeons, ACSM, American Medical Society for Sports Medicine, American Orthopaedic Society for Sports Medicine and American Osteopathic Academy of Sports Medicine. Representatives are chosen by their organisation based on their experience as team physicians with expertise in the topic area. The executive committee assigns select topics from the outline for the representatives who perform an evidence-based review of the existing literature. The outline is reviewed and modified by the executive committee and expert panel members and they then formulate statements that are supported by the literature and best practices into a format of ‘essential’ and ‘desirable’ information that the team physician is responsible for understanding. ‘Essential’ statements are information that every and any team physician^3^ must be responsible for understanding, whereas ‘desirable’ statements are those that are ideal, in the setting where every resource is available. TPCC papers are intended to provide general recommendations but are not meant to be prescriptive. The executive committee along with select expert consultant(s) collate and review the document over the course of 12–14 months, culminating in an in-person 2-day meeting of the executive committee and consultant(s) to finish compiling the paper into a rough draft. That meeting is followed by a 2-day meeting with all of the representatives during which the final paper is completed. This is a facilitated process where all topics of the paper are reviewed and exact wording is determined and agreed on. Consensus in this TPCC was reached by unanimous agreement. The final documents are then reviewed and approved by the board of directors of all six participating organisations.

**Outline: select topics in sport-related concussion**

- Definition.
- Biomechanics and pathophysiology.
- Epidemiology.
- Preseason planning and assessment.
- Same-day evaluation and treatment.
- Postsame-day evaluation and treatment|return-to-play.
- Diagnostic testing and management.
- Neurologic sequelae of brain injury.
- Persisting symptoms after sport-related concussion (PSaSRC).
- Prevention.
- Retirement/disqualification.
- Legislation and governance issues

### Definition

SRC is a traumatic brain injury, a pathophysiological process affecting the brain, induced by direct or indirect biomechanical forces (eg, a blow to the head or body).^4^

Common features include the following:

- Rapid onset of usually short-lived neurological impairment which typically resolves spontaneously.
- Acute signs and symptoms that reflect a functional disturbance rather than structural injury.
- A range of clinical symptoms that may or may not involve loss of consciousness (LOC).
- Routine neuroimaging studies (eg, traditional CT or MRI), if obtained are typically normal.
- Signs or symptoms that are not explained by other medical issues (eg, alcohol, drugs, medications, cervical spine injury, peripheral vestibular dysfunction, psychological disorder) or other co-morbidities.

### Biomechanics and pathophysiology

SRC occurs as a result of linear and/or rotational accelerations to the brain and can occur from either direct impact to the head or from transmitted (indirect) forces from the body to the head.

- Delineate the mechanism of injury.
  - Most commonly, these include head-to-head, head-to-body (eg, elbow, knee, shoulder), head-to-ground or head-to-object (eg, post, puck, stick).
  - Less commonly, these injuries involve transmitted or indirect forces (eg, whiplash).
  - The relationship between mechanism and signs, symptoms and severity is an area of emerging interest.
- Measured linear and rotational acceleration data alone do not accurately predict SRC.^5 6^
- No direct relationship between impact biomechanics and symptoms or cognitive performance change scores has been identified.^7^

Metabolic changes demonstrated in the animal model and thought to occur in humans include the following:

- Alterations in intracellular/extracellular glutamate, potassium and calcium.
- A relative decrease in cerebral blood flow in the setting of an increased requirement for glucose (ie, increased glycolysis). This mismatch in the metabolic supply and demand may potentially result in cell dysfunction and increase the vulnerability of the cell to a second insult. Changes in the inflammatory chemokines and mitochondrial function may also occur.^8 9^

There is presently insufficient evidence to correlate any single or combination of body fluid or imaging biomarkers as being diagnostic or prognostic for SRC.^10 11^

It is essential the team physician understand:

- SRC results from either direct impacts or transmitted forces.
- Symptoms of SRC are believed to result from changes in cerebral blood flow and an inability to match metabolic demands.

It is desirable that the team physician:

- Identify the mechanism of SRC.
- Understand there is currently no known threshold of linear or rotational acceleration to cause SRC.
- Understand there is insufficient evidence to correlate any single or combination of biomarkers as diagnostic or prognostic for SRC.

### Epidemiology

SRC commonly occurs in helmeted and non-helmeted sports and accounts for a significant number of time-loss injuries. There are limitations in SRC data (eg, injury definition, selection bias, reporting bias, incomplete surveillance).

Because of non-specific symptoms and lack of objective biomarkers, the true incidence and prevalence remains unknown. However, heightened awareness of and concern about SRC is associated with increased symptom reporting, which may not represent SRC.

Data from emergency department visits, office visits and a high school injury surveillance system (RIO) estimate 1–1.8 million SRCs per year in the USA in the age range of 0–18 years and a subset of about 400 000 SRCs in high school athletes.^12^

Published reports indicate:

- Rugby Union,^13^ American football, ice hockey, soccer, wrestling^14^ and lacrosse tend to have the highest concussion incidence rates when calculated by athlete exposure.
- Competition SRC rates are consistently higher than practice rates.
- In sports played with the same rules for males and females (eg, basketball, soccer, rugby), research suggests that the reported incidence rate of SRC is higher in female athletes.
- Data on age-related epidemiology are varied and conflicting.^15^
- The reported incidence of SRC is higher in high school and college athletes with a history of prior SRC, Attention Deficit Hyperactivity Disorder (ADHD) and/or learning disabilities.^16–19^

### Preseason planning and assessment

This is the period of the athletic year prior to any practice or competition. During this period, the emergency action plan (EAP) should be developed or reviewed and preparticipation assessment should be performed. EAP and assessments should include protocols and policies for recognition and acute management of SRC.

Each organisation should develop SRC protocols and policy including:

- Definition of SRC.
- Education
  - Recognising signs and symptoms of SRC.
  - Role of equipment.
  - Avoiding high-risk play behaviours (eg, leading with the head).
- Diagnosis
  - Only a licensed healthcare provider can make a SRC diagnosis.
- Acute sideline and post-injury management.
- Return to learn (RTL (academics)) and return to play (RTP) guidelines.

Athletes, coaches, parents, administrators, referees and healthcare providers should be educated about SRC. Education should occur in a manner consistent and compliant with state law, governing body and school district requirements.

Preparticipation assessment, including:

- Personal history
  - Prior SRC or brain injury.
  - Prior cervical spine injury
  - History of migraines
  - Seizure disorder and other neurological disease
  - Learning disability and ADHD
  - Depression or other mood disorders.
  - Medications, supplements, alcohol and drug use
- The utility of and necessity for baseline assessments is still being researched.
- Baseline assessments, when performed, include symptom checklist, cognitive function, balance/postural stability and other components of the neurological examination (eg, most-recent Sports Concussion Assessment Tool (SCAT) or child SCAT; neurocognitive testing (computerised and/or brief paper-and-pencil)).^20^

It is *essential* the team physician understand:

- The EAP, including guidelines specific to SRC management.
- The role of the preparticipation assessment as it relates to SRC.

It is *desirable* that the team physician:

- Practice and review the EAP, including recognition and acute management of SRC, at least on an annual basis.
- Perform a comprehensive preparticipation assessment including a personal history of SRC.
- Coordinate and be involved with baseline SRC assessment.
- Understand some SRCs may require a multidisciplinary team for care (eg, physician specialists, clinical psychologists, neuropsychologists, athletic trainers, school nurses, physical/occupational therapists), and these resources should be identified during the pre-season.

### Same-day evaluation and management

This is the period during or immediately after suspected SRC in practice or competition. Evaluation may occur on the field of play or sideline, off-field (eg, athletic training rooms) and should continue with serial evaluations.

#### On-field

- Immediate observable signs (studied in elite adult male athletes) have been shown to raise the index of suspicion for SRC include the following:
  - Lying motionless.
  - Motor incoordination (stumbling gait).
  - Tonic posturing (brief, non-sustained involuntary movements after trauma in an athlete with LOC; has been referred to as ‘impact seizure’).
  - Fall with no protective action (rag doll, floppy).
  - Blank/vacant look.
- Evaluate the injured athlete on-field in a systematic fashion:
  - Assess athlete’s level of consciousness (AVPU: alert, verbal, pain and unresponsiveness)
  - Assess airway, breathing, circulation
  - Perform a focused neurological assessment emphasising mental status, focal neurological deficit and cervical spine status (eg, on-field components of the SCAT and child SCAT).
  - Determine initial disposition (emergency hospital transport vs sideline evaluation).^20^ (table 1)

**Table 1:** Signs and symptoms requiring emergency hospital transport^20^

Red flags *Signs and symptoms requiring emergency hospital transport including:*	Immediate seizure (at or minutes after impact)^71^ More than brief LOC Severe or worsening headache Persistent or recurring emesis Deteriorating neurological status (eg, increasing lethargy, confusion) Persistent focal neurologic deficit (eg, tingling or paresthesias in extremities, diplopia) Cervical spine pain, bony tenderness, limited range of motion and/or deformity

LOC, loss of consciousness.

#### Sideline

- Obtain a more detailed history and perform a more detailed physical examination.
  - A distraction-free environment optimises the evaluation.
  - Assess for cognitive, somatic and affective signs and symptoms of SRC with particular attention paid to the number and severity of symptoms because of their prognostic significance.
  - Perform and repeat neurological assessments, with particular emphasis on cognitive function, cranial nerve and balance testing until the athlete is stable (eg, SCAT).
- Athletes with suspected SRC should be immediately removed from practice or competition.
  - If no licensed healthcare provider is present to evaluate the athlete with suspected SRC, there is no RTP even if symptoms resolve.
  - If SRC is confirmed by a licensed healthcare provider, there is no same-day RTP.
  - If evaluated by a licensed healthcare provider who determines a SRC did not occur, same-day RTP is an option.
- The athlete should not be left unsupervised until a disposition decision is made.
- Determine disposition for symptomatic and asymptomatic athletes, including serial assessments and postinjury follow-up (options include home with observation or transport to hospital).^20^
- Provide postevent instructions to the athlete and others (eg, medications, driving, alcohol, physical and cognitive exertion and medical follow-up).
- Final determination regarding SRC diagnosis and management is a medical decision based on clinical judgement.

It is essential the team physician:

- Know the signs and symptoms of suspected SRC.
- Recognise red flags (signs and symptoms and a protocol for emergency hospital transport).
- Know how to perform an on-field and sideline assessment that includes a neurological assessment (eg, SCAT and child SCAT).
- Understand athletes with suspected SRC should be immediately removed from practice or competition.
- Develop an initial disposition plan.
- Understand the determination of the diagnosis of SRC is a medical decision based on clinical judgement.

It is desirable the team physician:

- Perform an on-field and sideline assessment to include neurological assessment (eg, SCAT and Child SCAT)
- Participates in the same day assessment and management plan, including diagnosis, acute treatment and disposition.
- Educates the athletic care network (including certified and licensed athletic trainers, consulting physicians and other healthcare providers)^21^
- Prepare medical supplies for on-site rescue, immobilisation and transportation.^21^(table 2)

**Table 2:** Selected acute and delayed signs and symptoms suggestive of SRC

Cognitive	Somatic	Affective	Sleep disturbances
Confusion Anterograde amnesia Retrograde amnesia LOC Disorientation Feeling ‘in a fog’, ‘zoned out’ Vacant stare Inability to focus Delayed verbal and motor responses Slurred/incoherent speech Excessive drowsiness	Headache Dizziness Balance disruption Nausea/vomiting Visual disturbances (photophobia, blurry/double vision) Phonophobia	Emotional lability Irritability Fatigue Anxiety Sadness	Trouble falling asleep Sleeping more than usual Sleeping less than usual

LOC, loss of consciousness; SRC, sport-related concussion.

### Post-Same day evaluation and treatment

This is the period of time beginning the day after the injury and up to RTP. SRC is a treatable condition. The majority of athletes with SRC recover within a typical timeframe, currently defined as 2 weeks for adults and up to 4 weeks for children. The number and severity of the initial symptom burden is the best predictor for the duration of recovery after SRC.

During this time, symptom-specific interventions can be implemented while recovery is being monitored. It is also important that pre-existing, coexisting, and/or resulting comorbidities are addressed (eg, headache, anxiety, depression cervical spine pain).

Current evidence suggests strict rest after SRC slows recovery and increases the probability of prolonged symptoms.^22^ After a brief period of relative rest (24–48 hours), athletes may gradually and progressively resume cognitive and physical activity provided it does not produce new symptoms or exacerbate their existing symptoms. Recent studies have shown progressive moderate aerobic exercise within the first week helps safely speed recovery.^4^ Cognitive work should be modified or limited to that which does not produce or exacerbate symptoms.

#### Return to driving

No widely accepted return to driving protocols exist. Driving is a complex process involving coordination of cognitive, visual and motor skills, as well as concentration, attention, visual perception, insight and memory, which can all be affected by SRC. A decision on driving status should be part of the monitoring period.

#### Medication

Medications routinely prescribed prior to the SRC should be continued. Evidence of efficacy is limited and most athletes do not require the use of over-the-counter and/or prescription medications for acute SRC symptoms. In select situations where medication is considered, judicious use at the lowest dose for the shortest period of time is recommended. There is no current evidence that nutraceuticals are efficacious in the prevention or treatment of SRC.

#### Return to learn

**Figure 1:**
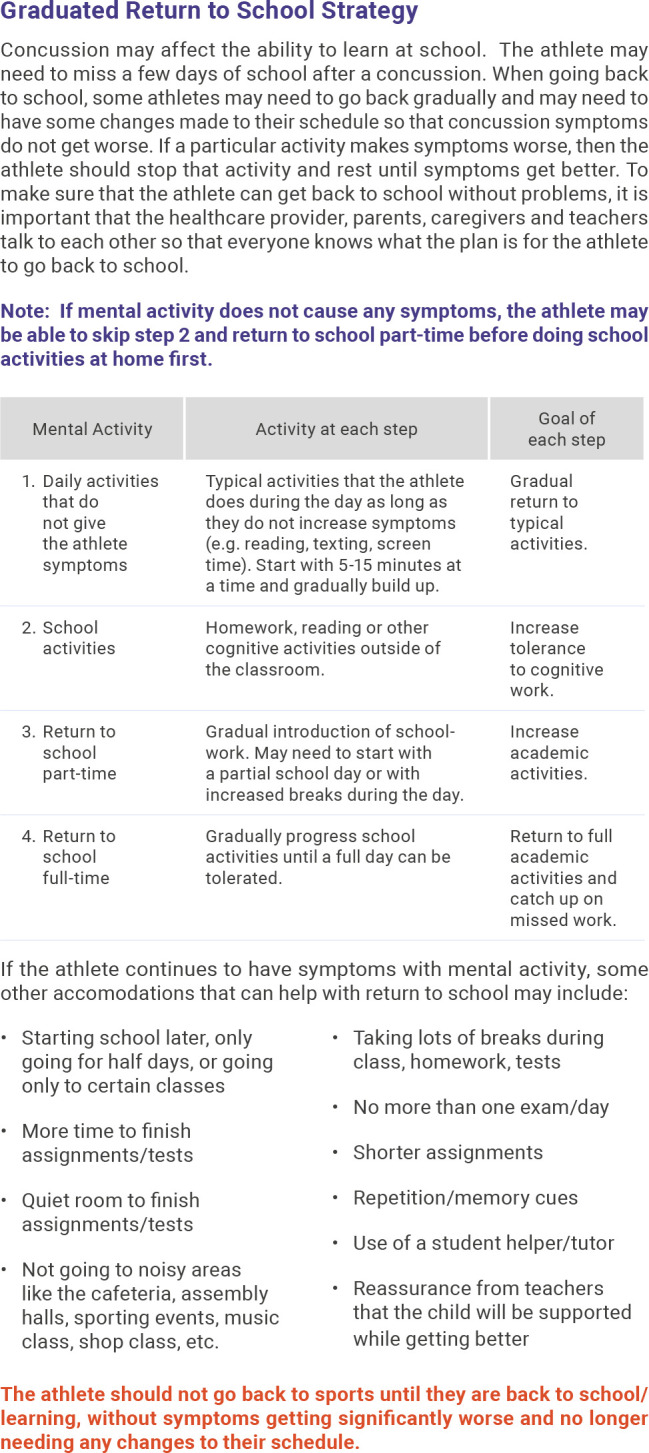
Return to learn.^20^

RTL (or return to class) is the process of transitioning back to class following SRC. Athletes should have regular medical follow-up after SRC to monitor recovery and help with return to class. Some athletes may require a short-term absence from or adjustment to their academic load after injury and then progressed as tolerated. The vast majority of athletes do not require prolonged absence from school or formal academic accommodations (eg, 504 Plan or Individualised Education Plan) after SRC. While RTL and RTP may progress simultaneously, successful completion of RTL precedes the final clearance for RTP (figure 1).^4^

#### Return to play

RTP is the process of transitioning back to practice and competition after SRC.^23^ Athletes should have regular medical follow-up after SRC to monitor recovery and help with RTP. RTP begins when athletes have reached their baseline level of symptoms, cognition and balance/postural stability.

**Figure 2:**
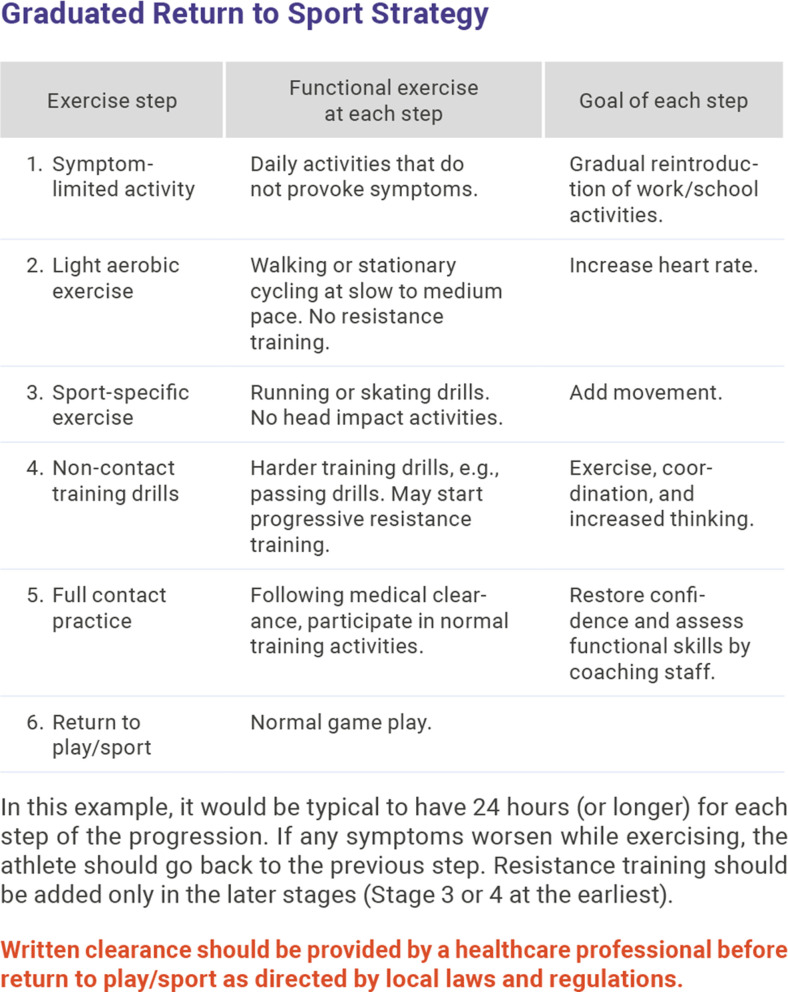
Return to play.^20^

Pharmacological therapy that has been prescribed for management of SRC symptoms must be considered carefully by the team physician. The decision to progress to and through RTP while on newly prescribed medication is made by the team physician. Progression involves a stepwise progression and increase in physical demands and sport-specific activities without return of symptoms before the final introduction of exposure to contact. The athlete also should demonstrate psychological readiness for RTP (figure 2).^24^

It is essential that the team physician understand:

- RTP is decided by the team physician.
- RTP follows a stepwise progression after an athlete has reached preinjury level of symptoms and function.
- RTL precedes final clearance for RTP.
- The majority of SRCs resolve in 2–4 weeks (varies by age).

It is desirable that the team physician:

- Provide the ongoing care and monitoring of the athlete with SRC.
- Participate in the RTL process.
- Coordinate the RTP process.
- Recognise that the number and severity of initial symptom burden is the best predictor for the duration of recovery after SRC.
- Understand that limited cognitive rest and early subsymptom threshold aerobic exercise is recommended for the treatment of SRC.

### Diagnostic testing

The diagnosis of SRC is clinical, and dependent on history, including mechanism of injury and physical examination. Some tests have been proposed as tools to aid in the clinical diagnosis. Other tests have been used for research purposes related to SRC. For each, more research is needed.

#### SCAT/Child SCAT

SCAT is a tool used to assist the team physician in evaluating SRC in young athletes 13 years old or older; Child SCAT is used to evaluate young athletes 12 years old and under. It is not a standalone tool for the diagnosis of SRC. SCAT is a multimodal tool, which includes validated components of Glasgow Coma Scale, Graded Symptom Checklist, Maddocks’ Questions, Standardised Assessment of Concussion (SAC) and the Modified Balance Error Scoring System. Utility of the SCAT significantly decreases 3–5 days postinjury.^20^ The tool is more clinically useful in the assessment of acute SRC, but has a limited role in tracking recovery except for the symptom checklist. Ceiling effects (high proportion of athletes’ scores at the highest level) were noted for the prior versions of SCAT, specifically the five-word Immediate Recall component of the SAC.^20^

#### Neurocognitive and neuropsychological testing

Formal neurocognitive tests include computerised neurocognitive and/or brief paper-and-pencil tests that assess cerebral functions (eg, attention/concentration, memory, learning, speed of information processing) affected by SRC. While not required, neurocognitive testing may provide additional useful information during the course of SRC and assist in making RTL and RTP decisions.^4^ The SAC, which is a brief neurocognitive test, is included in the SCAT.

Baseline or preseason neurocognitive testing is not obligatory but may be useful in the interpretation of post-SRC neurocognitive testing.^4^ With or without baseline neurocognitive testing, there may be a role for postinjury formal neurocognitive testing, particularly in cases of clinical uncertainty. Results of formal neurocognitive tests are best interpreted by a licensed, clinical neuropsychologist.^4^ These tests should not be the sole basis of SRC diagnosis and/or RTP decisions.

Formal neuropsychological testing is a more comprehensive assessment of cognitive functions, and also includes mood, developmental and personality measures and formal assessment of effort. This testing is conducted only by a licensed, clinical neuropsychologist and is best used in individuals with PSaSRC or pre-existing comorbidities.

#### Oculomotor

Impairment in oculomotor function may occur in SRC. Several tests have been designed to assess these specialised ocular functions, including saccadic eye movements and smooth pursuits, vestibular ocular reflex and near-point of convergence (NPC). To date, the research suggests that NPC may be the most useful oculomotor test.^25^

#### Spotters /video review

Methods have been identified for trained personnel (spotters) using live video with replay and slow-motion capabilities to recognise observable signs of suspected SRC.^26^ Spotters watch video of events/plays multiple times, at different angles and communicate with on-field team physicians about potential SRC. The data from the spotter/video review process have been shown to be helpful in SRC assessment of elite adult male athletes.^26^

#### Genetic testing

Genetic testing continues to be investigated as a method to identify risks for sustaining or experiencing prolonged recovery from SRC. However, there is no genetic test that is currently clinically useful for either purpose.

#### Neuroimaging

Neuroimaging has been shown to have little utility in the diagnosis of acute SRC but should be considered if there is concern for other head or neck injury (red flags).

Standard MRI can be considered in cases with persisting symptoms after SRC (PSaSRC). Advanced neuroimaging techniques (eg, MRI with diffusion tensor imaging, arterial spin labelling, quantitative susceptibility imaging remain research tools at this time.

#### Serum biomarkers

Several serum biomarkers are being investigated as objective measures of SRC. The most frequently studied include S100 Beta, ubiquitin carboxyl-terminal hydrolase Isoenzyme L1 (UCHL-1), Neuron Specific Enolase (NSE) and Glial Fibrillar Acidic Protein (GFAP). While there is some evidence that GFAP and UCHL-1 are helpful to identify an intracranial bleed, serum biomarkers are not currently indicated for identifying or managing SRC.^27 28^

#### Electrophysiological tests

Electrophysiological tests (EEG, evoked potentials) evaluate electrical activity in the brain. While this is an area of active research, there is currently not enough research to recommend utility in the diagnosis and treatment of SRC.^29^

It is *essentia*l that the team physician understand:

- The diagnosis of SRC remains a clinical diagnosis. There are tools and tests that may aid in its assessment but are not to be used as standalone measures.
- The indications for neuroimaging in athletes.

It is *desirable* the team physician understand:

- Formal neurocognitive testing is not required but can provide useful information for baseline and postinjury assessment, and aid in making RTL and RTP decisions.
- Indications for and limitations of neurocognitive and neuropsychological testing.
- The difference between neurocognitive and neuropsychological testing.
- Impairment in oculomotor function may occur in SRC.
- The data from the spotter/video review process have been shown to be helpful in elite adult male athletes.
- Serum biomarkers, genetic testing, advanced neuroimaging and electrophysiologic testing are research tools.
- The need to educate the athletic care network on the various testing paradigms that exist in the diagnosis of concussion.

### Neurological sequelae of brain injury

There are some medical conditions that may be of concern to the team physician and may or may not be related to SRC. These include the following:

- Concussive convulsion
- Second-impact syndrome/diffuse cerebral oedema
- Recurrent SRC
- Chronic traumatic encephalopathy (CTE).

#### Concussive convulsion

- Brief tonic phase followed by myoclonic or clonic jerking of the extremities within 2 s of concussion event and lasting <150 s.^30^
- Often referred to as ‘impact seizure’, but not epileptiform or true seizure.
- Associated with brief LOC.
- Reported incidence about 1/70 concussions.^31 32^
- Requires no specific management beyond on-field/sideline assessment.
- No neuroimaging or electroencephalography (EEG) is recommended.
- No anticonvulsant therapy is required.
- Not a significant risk factor for developing post-traumatic epilepsy.^30^

#### Second-impact syndrome (SIS)/diffuse cerebral oedema

A rare syndrome which appears to be caused when an athlete suffers a second (milder) blow to the head while still symptomatic from a recent head injury, including SRC, which is typically in the same sporting event.

- Nearly all cases have been reported in male athletes age 13–23 years old.^33^
- More needs to be understood about its pathophysiology.
- Vascular engorgement leads to a massive increase in intracranial pressure and brain herniation resulting in severe brain damage or death.
- Its catastrophic outcome underscores the importance of and can limit the risk of SIS by:
  - Educating athletes about accurate and immediate symptom reporting.
  - Immediately removing athletes with suspected SRC from practice or competition until evaluated by a licensed healthcare provider.
  - Complete clinical recovery before RTP.

#### Recurrent concussion

Sustaining a diagnosed SRC is a risk factor for another diagnosed SRC. However, there is high variability and reasons are multifactorial.^34 35^

- Complete clinical recovery from the first SRC diminishes the risk of recurrent SRC.
- Athletes with recurrent SRC may or may not have prolonged recovery with a subsequent SRC.
- A history of SRC should be recognised during the preparticipation evaluation including number, recovery course and time between injuries.

#### CTE

CTE is defined as a delayed onset, distinct, progressive neurodegenerative disease (tauopathy) reported to be caused by repetitive brain trauma.^36^

The incidence, prevalence and pathophysiology of CTE is unknown. Although clinical diagnostic criteria have been proposed,^37 38^ CTE is diagnosed only via autopsy and is based on a preliminary and singular neuropathologic criterion.^36^ Initial signs and symptoms do not typically manifest until midlife^39^ or decades after exposure to trauma,^40^ and include a significant decline in cognition (recent memory, executive function), mood (depression, anger, irritability) or behaviour (impulsivity, aggressiveness, suicidal behaviour) with eventual progression to dementia.^37 38^

Current evidence does not indicate a higher risk of CTE in youth athletes who sustain multiple SRC.^41^ ‘High quality data show no association between repetitive head impact exposure in youth and long-term neurocognitive outcomes’.^42^

CTE pathology has been studied primarily in boxers and American football players and has been found in athletes from some other sports as well as in other medical conditions.^43^

CTE is an important topic that warrants further study, and prospective longitudinal population-based studies are needed.

It is essential that the team physician:

- Mitigate neurological sequelae of brain injury.
- Understand there is not a higher risk of developing CTE in youth athletes who sustain a single or multiple SRC.
- Understand SIS is a rare but catastrophic outcome of SRC.

It is desirable that the team physician:

- Understand sustaining a diagnosed SRC is a risk factor for diagnosis of another SRC.
- Understand concussive convulsions are not a risk factor for developing CTE.
- Counsel the athletic care network about conditions that may be related to SRC, and the potential significance of the long-term consequences.

#### Persisting symptoms after SRC

The majority of athletes with SRC recover within the typical timeframe, currently defined as 2 weeks for adults and up to 4 weeks for children. *PSaSRC* is defined as symptoms that last longer than the typical timeframe.

The pathophysiology underlying PSaSRC is not entirely understood. It is thought PSaSRC is not caused by a single pathologic process, but rather an interaction of postinjury symptoms that are complicated by pre-existing, coexisting and/or resulting biopsychosocial factors.^4 29 44^

It is important to perform a thorough evaluation for other etiologies of the symptoms, such that these may be identified and treated appropriately.

Factors that may be associated with greater odds of developing PSaSRC include:

- High symptom load immediately after injury
- Prolonged strict cognitive and physical rest (more than a few days) after the concussion
- Unrecognised, untreated or inadequately treated initial SRC.
- Suffering another direct impact to the head or from transmitted (indirect) forces from the body to the head while still symptomatic from a concussion.
- Personal or family history of migraine or mood disorder
- Premorbid diagnosis of learning difficulties
- Family and social stressors
- Increased baseline level of SRC symptoms (pre-SRC)

Of all these risk factors, pre-existing mood disorder and high symptom load immediately after injury have been shown to be the most consistent predictors of PSaSRC.^26 32^

Athletes who are removed from activity immediately after the injury seem to be less likely to develop PSaSRC than those who continue to play immediately after the injury.^45 46^

Risk for PSaSRC may be reduced by removing the athlete from play immediately after the injury, instituting relative physical and cognitive rest for the first 24–48 hours, and then gradually resuming usual activities that do not exacerbate symptoms.^46^

Early individualised subsymptom threshold aerobic exercise training may reduce the risk for developing PSaSRC.^47^

### Treatment of persisting symptoms after SRC

With treatment(s), most athletes with PSaSRC will recover without any long-term sequelae. A small percentage may experience long-term symptoms or deficits.

Athletes with PSaSRC require symptom-targeted treatment which may involve a multidisciplinary team.^29 44 48^ Treatments that have been shown to be effective in facilitating recovery of specific symptoms for athletes with PSaSRC include cervical spine rehabilitation, subsymptom threshold aerobic exercise training, vestibular and oculomotor therapy, cognitive behavioural therapy, academic adjustments and lifestyle adjustments involving sleep, nutrition and hydration.^48–56^ Pharmacologic treatment is used rarely in SRC and selectively for PSaSRC.

#### Fatigue

Fatigue is a common PSaSRC. Etiologies can be multifactorial and require a comprehensive evaluation. Treatment should be targeted to the suspected primary cause (eg, SRC, poor sleep, depression, nutritional deficiency, infection).

#### Mental health issues (anxiety/mood)

Mood changes are a common PSaSRC. Etiologies can be multifactorial and require comprehensive evaluation. Evaluation should include validated tests, examples of which include the Beck Depression Inventory II, Beck Anxiety Inventory, Patient Health Questionnaire 9, Generalised Anxiety Disorder 7 and Paediatric Symptom Checklist. A referral to a licensed mental health practitioner may be indicated.

#### Headache/migraine

Headache is a common PSaSRC. Persistent headaches occur in 10%–30% of athletes after a SRC.^4 57 58^ Athletes with persisting headaches or migraines should be evaluated for pre-existing or underlying disorders that may be causing or contributing to headache.

Treatment should be targeted towards the underlying mechanisms (eg, migraine disorder, unresolved vestibular or visual deficits, dehydration, injury or dysfunction in the upper cervical spine) and may require multidisciplinary care. Medication-overuse headaches can be caused by long-term use of nonsteroidal anti-inflammatory medications.^59^

#### Sleep disturbance

Sleep disturbances may pre-exist, coexist with and/or result from SRC and may include insomnia, hypersomnolence or hyposomnia. Disordered sleep may have adverse effects on cognitive function, mood and pain perception and delay recovery from SRC. Etiologies are multifactorial and warrant a thorough evaluation. Pharmacologic and non-pharmacologic treatment strategies (eg, medication, sleep hygiene including screen time management) should be considered. A sleep medicine specialist referral may be considered.

#### Vestibular/oculomotor

Persisting oculomotor symptoms may occur, and pre-exist, coexist with and/or result from SRC. Oculomotor symptoms are detected through history and physical examination including validated oculomotor tests. Mitigating risk factors include pre-existing vestibular dysfunction (eg, motion sickness). Specialists including vestibular rehabilitation therapist, otolaryngologist and audiologist may be considered.

It is essential that the team physician understand:

- Most athletes with SRC recover within the typical timeframe.
- PSaSRC is defined as symptoms lasting beyond the typical recovery time.
- Immediate removal from play after a concussion may decrease the risk for PSaSRC.

It is desirable that the team physician understand:

- Athletes with PSaSRC should have a thorough evaluation for other etiologies of their symptoms.
- Prolonged strict physical and cognitive rest are not recommended because it increases the risk for PSaSRC.
- Treatment for athletes with PSaSRC is symptom-targeted and may require a multidisciplinary team.
- Pharmacologic treatment is used rarely in SRC and selectively for PSaSRC.
- The most common risk factors associated with developing PSaSRC include a pre-existing mood disorder and high symptom load immediately after injury.
- The various symptom-targeted treatments shown to be effective for PSaSRC.
- Early individualised subsymptom threshold exercise training may reduce the risk for developing PSaSRC.

### Prevention

Primary prevention of SRC is not completely possible.

- Helmets do not prevent all SRC, although they decrease the incidence of skull fracture and moderate-to-severe traumatic brain injuries; in professional American football players, it has been reported that certain helmet models are associated with a lower rate of SRC.^60^
- There is currently no evidence to support the use of headgear or helmets in sports to prevent all SRC.^61–66^
- Headgear in soccer, rugby, wrestling, boxing may decrease the risk of lacerations and soft tissue trauma.^61–66^
- Improper use of the head in sport-specific skills, and improper fit of a helmet or required protective equipment may increase the risk of SRC.
- Mouth guards may decrease the risk of dental or oral injury; more research is needed to validate their use in limiting risk of SRC.

There are strategies that may be helpful in primary prevention of SRC, including:

- Limiting contact drills in practice.
- Teaching sport-specific technique (eg, blocking, tackling checking).
- Fitting of equipment, with more research needed to validate their role in limiting risk of SRC.
- Enforcement of rules that prohibit hits to the head and other potentially unsafe conduct (eg, spearing, leading with the head).
- Rule changes (eg, checking in youth ice hockey, kickoff return, blindside blocks, targeting and limited pyramid height in cheerleading).
- ‘Fair Play’ (rewarding proper behaviour and penalising improper behaviour in ice hockey played in youth).^67^
- More research is needed to recommend cervical spine strengthening and neuromuscular training as primary prevention strategies.

Prevention of disability associated with SRC is an important goal. Strategies include the following:

- Immediate recognition and removal from practice and play.
- Avoid prolonged strict cognitive and physical rest.
- Complete clinical recovery after SRC before RTP.
- In PSaSRC situations, a multidisciplinary team should be considered for evaluation and management, particularly in athletes who are significantly disabled.
- Education for the athlete and other members of the athletic care network about the signs and symptoms of SRC and the importance of immediate recognition and reporting.

It is essential the team physician understand:

- SRCs are not completely preventable.
- In PSaSRC situations, a multidisciplinary team should be considered for evaluation and management, particularly in athletes who are significantly disabled.

It is desirable the team physician:

- Understand strategies and interventions that may minimise risk of SRC and PSaSRC.
- Educate the athletic care network regarding prevention strategies.(table 3)

**Table 3:** Risk factors that may prolonger complicate recovery from SRC

Factors	Sentence
Concussion History	Total number, proximity, severity (duration)
Symptoms	Total number, severity (intensity and especially duration)
Signs	Prolonged LOC (>1 min)
Susceptibility	Concussions occurring with lower impact magnitude and/or requiring longer recovery
Age	Youth and adolescent athletes may recover more slowly
Pre-existing conditions	Migraine, attention deficit hyperactivity disorder (ADHD), learning disabilities (LD), depression, anxiety/panic attacks, motion sickness/sensitivity

LOC, loss of consciousness; SRC, sport-related concussion.

### Legislation and governance issues

All 50 states in the USA and the District of Columbia have passed youth SRC laws, and recurrent SRC rates have been declining.^68^

These laws have been further modified in many states to include topics such as RTL, practice modifications and expansion of coverage to more groups of healthcare providers and athletes. Similar legislation has been enacted in Ontario, Canada.^69^

In addition to state laws, local, national and international^70^ governing bodies have continued to adopt rule changes and develop guidelines. The team physician is affected by legislation and governance issues both administratively and clinically. As research continues, opportunities exist to further refine these laws and guidelines to optimise efficacy.

It is essential that the team physician understand:

- The laws of the country and/or state in which they are practicing regarding SRC.
- Rules and regulations from governing bodies regarding SRC.

It is desirable the team physician:

- Participate with state athletic associations in advocacy (eg, interscholastic associations)
- Participate in the education of the athlete, parents/guardians, coaches, caregivers and others.

### Retirement and disqualification

Retirement decisions from contact or collision sports should typically be a shared decision involving the athlete, parents, guardians, healthcare providers and others based on multiple factors, including the risks and benefits of continuing participation in the sport.^42^

There is no clear evidence regarding a specific number of SRC in youth sports that mandates retirement/disqualification.

There is limited evidence regarding whether the discontinuation or continuation of participation in a youth sport is associated with long-term brain health and well-being. There are relative contraindications to continuing to play a contact or collision sport. These include trauma-related structural brain abnormalities identified on neuroimaging, persistent neurological abnormalities on physical examination, PSaSRC, SRC symptoms occurring with lesser impacts, permanent deficit on neuropsychological testing, psychological readiness to RTP and/or retire and prior history of intracranial haemorrhage, arachnoid cyst, Chiari malformation.

More research is needed to better determine evidence-based criteria for retirement/disqualification of youth athletes after SRC and/or repeated head trauma.

It is essential that the team physician understand:

- There is not a specific number of SRC that automatically lead to retirement/disqualification.
- Retiring from youth sports after SRC is often best addressed via a shared decision-making model.
  It is desirable that the team physician:
- Understand the relative contraindications to continuing participation in contact or collision sports after SRC.
- Be involved in making retirement/disqualification decisions.
- Participate in the education of the athlete, parents/guardians, coaches, healthcare providers and others

### Limitations

The Team Physician Consensus Statement published series is not intended as a standard of care, and should not be interpreted as such. This document is only a guide, and as such, is of a general nature, consistent with the reasonable, objective practice of the healthcare professional. The focus populations for the statement are those individuals that a team physician would care for, typically the child to college or Olympic level aged athlete. Physician representatives from North America comprised the writing group. Given the broad nature of topics, only select topics are included. In addition, formal systematic review of the literature and level of evidence statements or strength of recommendation taxonomy are not included.

The opinions and assertions expressed herein are those of the author(s) and do not necessarily reflect the official policy or position of the Uniformed Services University or the Department of Defence or any of the individual institutions or leagues that authors are affiliated with.

### Executive committee

Stanley A Herring, MD., Chair, Seattle, Washington.

W Ben Kibler, MD., Lexington, Kentucky.

Margot Putukian, MD., Princeton, New Jersey

### Consultant

Gary Solomon, PhD, Colchester, VT

### Expert panel

Lori A Boyajian-O’Neill, DO Overland Park, KS.

Katherine L Dec, MD., Richmond, VA.

R Robert Franks, DO, Marlton, NJ

Peter A Indelicato, MD, Gainesville, FL.

Cynthia R LaBella, MD, Chicago, IL

John Leddy, MD, Buffalo, NY

Jason Matuszak, MD, Buffalo, NY

E Barry McDonough, MD, Morgantown, WV

Francis G. O’Connor, MD, Bethesda, MD.

Karen M Sutton, MD, New York, NY
